# 4081 Quantifying pH buffering capacity and kinetics of tumor and healthy tissue to understand and exploit differences in biology

**DOI:** 10.1017/cts.2020.89

**Published:** 2020-07-29

**Authors:** A. Colleen Crouch, Emily A. Thompson, Mark D. Pagel, Erik N.K. Cressman

**Affiliations:** 1 The University of Texas Health Science Center at Houston; 2 University of Texas MD Anderson Cancer Center

## Abstract

OBJECTIVES/GOALS: The purpose of this work is to investigate natural buffering capacity of liver tissue and tumors, to understand and exploit differences for therapy. Using this work, we will determine the concentrations of reagents (acids or bases) used in ablation treatment to optimize treatment by increasing tumor toxicity and minimizing healthy tissue toxicity. METHODS/STUDY POPULATION: For this preliminary study, two methods will be used: benchtop pH experiments *ex vivo* and non-invasive imaging using acidoCEST MRI *in vivo*. For *ex vivo*, two types of tissues will be tested: non-cancerous liver and tumor tissue from HepG2 inoculated mice (n = 10). After mice are euthanized, pH will be measured in tissue homogenates at baseline and then the homogenates will be placed in either acidic (acetic acid) or basic (sodium hydroxide) solutions with varied concentrations (0.5–10M) and time recorded until pH returns to baseline. For *in vivo* imaging, Mia PaCA-2 flank model mice (n = 10) will be imaged with acidoCEST MRI to quantify pH at baseline. Mice will then be injected intratumorally with (up to 100 μL of) acid or base at increasing concentrations and imaged to quantify pH changes in the tumor. RESULTS/ANTICIPATED RESULTS: For this study, buffering capacity is defined as the concentration threshold for which tissue can buffer pH back to within normal range. Non-cancerous tissue is likely to buffer a wider range of concentrations compared to tumor tissue. From the benchtop experiment, comparison of time-to-buffer will be made for each concentration of acid/base for the two tissue types. AcidoCEST MRI will provide *in vivo* buffering capacity and potentially demonstrate tumor heterogeneity of buffering capacity. For both experiments, a pH vs. concentration curve for the two tissue types will allow for comparison of ex vivo to *in vivo* experiments, which will differentiate contributions of local tissue buffering capacity from the full body’s natural bicarbonate buffer system that depends on respiration and blood flow. DISCUSSION/SIGNIFICANCE OF IMPACT: The pH of the body must be maintained within a narrow range. With cancer, impairment in regulation of tumor metabolism causes acidosis, lowering extracellular pH in tumors. It remains unclear if pH plays a role in local recurrence or tumor toxicity. This work will determine if acidoCEST MRI can measure deliberate alteration of pH and how this change affects biology.

